# Reply

**DOI:** 10.1016/j.jvsv.2026.102466

**Published:** 2026-06-22

**Authors:** Shuai Tang, Kai Li, Fan Chang, Song Li, Zheng Lv, Jianghui Zhang, Wensong Wu, Huiyuan Shi, Fangmin Chen

**Affiliations:** Department of Urology, Tianjin Third Central Hospital, Tianjin, China; College of Medicine, Nankai University, Tianjin, China; Department of Urology, Tianjin Third Central Hospital, Tianjin, China; Department of Urology, Wuhan Sixth Hospital, Wuhan, China; Department of Urology, Tianjin Third Central Hospital, Tianjin, China; Department of Urology, Tianjin Third Central Hospital, Tianjin, China; Department of Urology, Wuhan Sixth Hospital, Wuhan, China

We thank the correspondents for their thoughtful comments on our retrospective single-center series of laparoscopic and robot-assisted extravascular stenting (EVS) for anterior nutcracker syndrome (NCS). We appreciate the opportunity to clarify the intent, scope, and limitations of our proposed postoperative duplex ultrasound targets and the five-in-a-row operative checklist.

First, we fully agree that deriving and assessing a hemodynamic cutoff within a small cohort can yield optimistic estimates of discrimination and an internally best threshold that may not generalize. Accordingly, we do not present an aortomesenteric peak systolic velocity of ≤72 cm/s as a universal gold standard. Rather, it is a unified, reproducible primary attainment end point observed under a standardized single-center workflow and follow-up paradigm, intended to support consistent postoperative assessment and hypothesis generation. As stated in our Limitations and Conclusion, the rarity of NCS and availability of conservative options constrained sample size and statistical power, and the small sample precluded robust adjustment methods (eg, propensity score matching) without compromising statistical stability; approach selection was nonrandomized with baseline imbalances, so between-approach comparisons are exploratory only.^1^ We, therefore, concur that the appropriate next step is external validation of the aortomesenteric peak systolic velocity of ≤72 cm/s target in larger, multicenter cohorts with standardized duplex ultrasound protocols, with attention to calibration, learning curve effects, and operator variability.

Second, we agree that the five procedural elements are evaluated as a bundled intervention in this retrospective analysis, and that the independent mechanistic weight of each step cannot be disentangled without prospective designs. Our goal was to address a practical problem in EVS—heterogeneity in intraoperative execution—by providing a transparent process framework that can be reproduced and audited. With respect to tributary management, our inference was hypothesis generating: in a small subset with incomplete/relapsing clinical success despite imaging improvement, incomplete tributary control was a recurring intraoperative feature. We acknowledge that this observation cannot establish causality and requires confirmation in larger datasets with granular intraoperative fidelity metrics.

Finally, we share the correspondents' interest in the possibility that venous hypertension may mediate renal parenchymal injury and that symptom trajectories may not perfectly align with early hemodynamic shifts. Consistent with this, we were cautious to limit our clinical conclusions to the available follow-up window and to note that postoperative goals may help to predict outcomes, but do not provide real-time intraoperative correction guidance. Supporting the biological plausibility of delayed or incomplete recovery, our experimental rat NCS model suggested that sustained renal venous congestion can precipitate marked renal injury and fibrosis within days.^2^ Ongoing work will focus on longer-term, patient-centered follow-up integrating serial patent-reported outcomes, imaging, and physiology to define durability after EVS.

In sum, we welcome the dialogue and emphasize that our proposed targets and checklist are intended to standardize postoperative assessment and procedural reproducibility while motivating multicenter validation and mechanistic dissection.

## Funding

None.

## Disclosures

None.
